# Case Report: Pharmacogenetically-triggered fatal serotonin syndrome following sequential serotonergic therapy in Parkinson’s disease: a case highlighting the role of CYP2D6*10/*10 genotype and multifactorial drug interactions

**DOI:** 10.3389/fpsyt.2026.1750596

**Published:** 2026-03-31

**Authors:** Dong-Yang Liu, Ya-Hui Yi, Na Li, Fang-Ming Luo, Wei-Jing Gong

**Affiliations:** 1 The Eighth People’s Hospital of Longgang District, Shenzhen, Guangdong, China; 2 Department of Pharmacy, Union Hospital, Tongji Medical College, Huazhong University of Science and Technology, Wuhan, China

**Keywords:** CYP2D6, drug interaction, rasagiline, selective serotonin reuptake inhibitors, serotonin syndrome

## Abstract

Serotonin syndrome (SS) is a potentially life-threatening condition resulting from excessive serotonergic activity in the central nervous system. We present a fatal case of SS complicated by multiple organ failure in a 72-year−old patient with Parkinson’s disease following the sequential administration of two selective serotonin reuptake inhibitors (sertraline and escitalopram) while on a stable regimen of the monoamine oxidase-B inhibitor rasagiline. Pharmacogenomic testing revealed a homozygous CYP2D6*10/*10 genotype, conferring an intermediate metabolizer phenotype, which is postulated to have contributed to serotonergic drug accumulation and resultant toxicity in combination with other significant pharmacodynamic and pharmacokinetic interactions. This case was distinguished by three key features: first, the sequential serotonergic challenge from two different SSRIs in combination with rasagiline; second, the unprecedented severity of clinical manifestations, including rhabdomyolysis, acute hepatic and renal injury, and a disseminated intravascular coagulation-like state; and finally, the pharmacogenetic findings that provide a partial mechanistic explanation for the extreme drug sensitivity. This report underscores the critical importance of pre-emptive pharmacogenomic screening in patients receiving complex polypharmacy, particularly when combining drugs with serotonergic properties. It also serves as a critical warning that even selective MAO-B inhibitors can precipitate life-threatening interactions with SSRIs in genetically susceptible individuals, thereby informing more stringent personalized therapeutic strategies.

## Introduction

Serotonin syndrome (SS) is a potentially life-threatening condition resulting from excessive stimulation of central and peripheral serotonin receptors. The classic clinical triad encompasses autonomic dysfunction, neuromuscular excitability, and altered mental status (1). The increasing global use of serotonergic agents, particularly selective serotonin reuptake inhibitors (SSRIs), has led to a corresponding rise in the incidence of SS (1). The primary pathophysiological mechanism involves drug-induced elevation of synaptic serotonin levels, which, in severe cases, can escalate to hyperthermia, rhabdomyolysis, and multi-organ failure (2).

The risk of SS is substantially amplified by pharmacodynamic interactions, especially the concomitant use of multiple serotonergic drugs. The combination of SSRIs with monoamine oxidase inhibitors (MAOIs) represents a well-documented and particularly hazardous interaction (3). Although rasagiline is a selective MAO-B inhibitor with theoretical safety advantages, accumulating evidence confirms that its combination with SSRIs can still precipitate severe serotonin-related toxicity (4). Beyond pharmacodynamic interactions, interindividual variability in drug metabolism plays a critical role. Genetic polymorphisms in cytochrome P450 enzymes, notably *CYP2D6, CYP2C19*, can result in intermediate or poor metabolizer phenotypes, significantly increasing the systemic exposure to substrates like SSRIs and thereby exacerbating the risk of severe adverse drug reactions (5).

Despite these known risks, the specific scenario of sequential administration of two different SSRIs (sertraline followed by escitalopram) in a patient stabilized on rasagiline remains poorly characterized in the literature. Furthermore, the compounding effect of a *CYP2D6* intermediate metabolizer genotype (CYP2D6*10/*10) in such a context has not been previously detailed and the contribution of other metabolic pathways (e.g., CYP3A4) to a complex drug-drug interaction network is often overlooked. This case report addresses this knowledge gap by demonstrating how standard doses of serotonergic drugs can culminate in life-threatening SS in a genetically susceptible individual who was also exposed to a complex regimen of multiple CYP3A4 substrates and inhibitors. By integrating detailed clinical narrative with pharmacogenomic analysis, this report underscores the imperative of pre-emptive genetic testing in guiding safe polypharmacy, especially in complex patients requiring multi-drug regimens.

## Case presentation

### Patient information

A 72-year-old Chinese male with a history of Parkinson’s disease for more than six years (Hoehn and Yahr stage 4) was admitted for management of motor fluctuations on August 4, 2025. His pre-admission pharmacological regimen was complex and included benserazide/levodopa (187.5 mg tid), carbidopa/levodopa controlled-release (125 mg qd), and rasagiline (1 mg qd). Concomitant medications for comorbidities comprised rivaroxaban (10 mg qd), rosuvastatin (10 mg qd), memantine (20 mg qd), and quetiapine fumarate (62.5 mg qn). His past psychiatric history was significant for a single depressive episode five years prior, which was successfully treated with sertraline 50 mg daily for one year without adverse effects. There was no history of substance abuse or suicidality. Vital signs at admission were stable. However, in May 2025—approximately five months prior to the October psychiatric consultation—a re-exposure to sertraline (prescribed by an outside provider for recurrent depressive symptoms, dose and duration unclear) was associated with the development of mild tremor and agitation. Critically, these symptoms were subtle and difficult to distinguish from his underlying Parkinson’s disease motor fluctuations; they resolved upon discontinuation of sertraline but were not recognized at the time as possible prodromal serotonergic hyperactivity.

### Clinical course and timeline

The patient’s hospital course remained stable until mid-October 2025, when he exhibited significant neuropsychiatric deterioration. During this period, the patient was hospitalized in the Rehabilitation Department, with clinical management provided by the Neurology team and psychiatric consultation obtained as needed. On October 17, a psychiatric consultation was conducted. The patient was alert but displayed impulsive behavior, which had increased in frequency over the preceding two weeks. He also reported persistent fatigue and depressed mood, refusing to participate in therapy. His Hamilton Depression Rating Scale (HAMD-17) score was 19, and his Hamilton Anxiety (HMA) score was 7. The clinical impression was that the impulsive behavior might be related to dopaminergic therapy, leading to a dose reduction of benserazide/levodopa to 125 mg tid. To address the prominent depressive symptoms, the consulting psychiatry team initiated sertraline 50 mg daily on October 16.

On October 18, after two doses of sertraline, the patient developed unequivocal signs of serotonergic hyperactivity, including facial flushing, bilateral tremor, tachypnea, and labile hypertension (peaking at 157/89 mmHg). Given the clear temporal association and his documented history, sertraline was identified as the precipitant and was discontinued after the evening dose on October 17.

**Figure 1:**
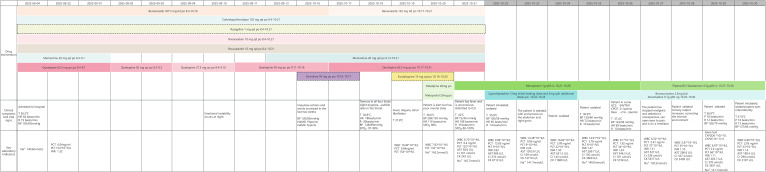
Timeline of drug administration and clinical course of serotonin syndrome.

In a pivotal and consequential subsequent decision, the consulting psychiatry team switched the antidepressant therapy to escitalopram 10 mg daily on the morning of October 19, without allowing for an adequate pharmacokinetic washout period. This initiated a catastrophic clinical cascade: the patient continued to experience facial flushing and tremor. By 16:15 on October 19, a low-grade fever of 37.6 °C developed. This escalated to marked hyperthermia (38.6 °C) and a hypertensive crisis (208/130 mmHg) by October 20. On the evening of October 20, the patient became increasingly agitated. The situation culminated on October 21 by late morning with a fulminant presentation of unresponsiveness and pupillary abnormalities (left 3.0mm, reactive; right 2.0mm, sluggish). This critical state necessitated emergency transfer to the intensive care unit (ICU). Upon ICU admission shortly thereafter, the patient was in a deep coma (Glasgow Coma Scale E1V1M1), and soon developed respiratory distress with oxygen desaturation to 85%. Serial axillary temperature measurements confirmed the severity of hyperthermia, which rapidly rose from 39.5 °C to a peak of 42.0 °C within approximately one hour, before declining to 41.5 °C by early afternoon. Concurrently, the patient exhibited progressive pupillary abnormalities: that evening, his pupils were unequal (left 3.0mm, right 2.0mm) with sluggish left and absent right light reflexes; by the following day (October 22), both pupils were unreactive to light, with observed scleral icterus. **Figure 1** provides a detailed timeline of the patient’s clinical course, juxtaposing key medication changes with the evolution of symptoms and organ dysfunction.

### Diagnostic assessment

The diagnosis of severe serotonin syndrome was unequivocal, fulfilling the Hunter Serotonin Toxicity Criteria with high specificity. The application of these criteria to the patient’s clinical findings is detailed in **Supplementary Table 1**.

Critical differential diagnoses were rigorously excluded. Serum sodium levels, measured using the indirect ion-selective electrode method, were repeatedly within the normal range (136–146 mmol/L), effectively ruling out SSRI-induced hyponatremia as a confounder. Neuroleptic malignant syndrome was considered unlikely due to the absence of “lead-pipe” rigidity, the presence of hyperreflexia and myoclonus, and the fact that dopaminergic therapy was not abruptly withdrawn. The initial presentation of fever preceding a significant rise in procalcitonin and other inflammatory markers argued against septic shock as the primary inciting event, though a subsequent hospital-acquired pneumonia (Klebsiella pneumoniae) was diagnosed during the ICU stay.

Pharmacogenomic analysis provided the mechanistic cornerstone for the patient’s extreme susceptibility. Testing revealed a CYP2D6*10/*10 homozygous genotype. According to the current Clinical Pharmacogenetics Implementation Consortium (CPIC) guidelines, this genotype corresponds to an activity score of 0.5 and is classified as an intermediate metabolizer phenotype (5). The analysis also revealed a CYP2C19*1/*2 heterozygous genotype, consistent with an intermediate metabolizer phenotype. This genetic profile created a high-risk pharmacokinetic milieu for the accumulation of both SSRIs.

### Therapeutic measures

Management in the ICU required a multidisciplinary, escalation-based approach: 1) Immediate Cessation: All serotonergic agents were withdrawn. 2) Aggressive Supportive Care: This included vigorous external cooling, vasopressor support with norepinephrine, and rhythm control with amiodarone. The profound coagulopathy (platelet nadir 9×10^9^/L, INR 3.45, fibrinogen 0.87 g/L) suggestive of disseminated intravascular coagulation mandated aggressive transfusion support. 3) Specific Antagonism: Cyproheptadine, a 5-HT2A receptor antagonist, was administered via nasogastric tube. An initial loading dose of 12 mg was given at 17:00 on October 22, followed by 2 mg every two hours from 19:00 on October 22 until 16:00 on October 24, for a total of 21 additional doses. The cumulative dose was 54 mg. 4) Organ Support: Continuous renal replacement therapy (CRRT) was instituted to manage refractory hyperthermia, acute kidney injury (creatinine 212 μmol/L), and acid-base derangements. 5) Complication Management: Antimicrobial therapy was escalated to meropenem and later to piperacillin-tazobactam based on rising procalcitonin and subsequent culture data. Bromocriptine (2.5 mg bid) was added during the recovery phase to address potential dopaminergic hypoactivity.

### Follow-up and outcome

After 10 days of intensive organ support, the patient’s hemodynamic and inflammatory parameters stabilized. However, his neurological status showed only marginal improvement. Upon ICU admission (Day 1), his Glasgow Coma Scale (GCS) score was E1V1M1. By Day 6, this had improved to E4VTM1, and by Day 9, to E4VTM2, indicating persistent severe brain injury with some brainstem recovery. Brain magnetic resonance imaging (MRI) performed on day 5 of the ICU stay revealed diffuse anoxic-ischemic encephalopathy, predominantly affecting the basal ganglia and watershed areas, with no evidence of intracranial hemorrhage or acute infarction. He remained in a comatose state (GCS E4VTM1) with persistent renal failure (creatinine 290 μmol/L). Following a family conference regarding the grave neurological prognosis, the patient was transferred to a long-term care facility, representing an outcome of severe disability. The severe neurological damage was thus clinically defined as a persistent comatose state with imaging findings consistent with profound hypoxic-ischemic injury.

## Discussion

This report delineates a fatal trajectory of serotonin syndrome (SS), unequivocally demonstrating how pharmacogenetic predisposition can converge with multi-drug therapy to precipitate catastrophic outcomes. The case compels a re-evaluation of therapeutic complacency in polypharmacy, particularly concerning the interplay between monoamine oxidase-B (MAO-B) inhibitors, selective serotonin reuptake inhibitors (SSRIs), and genetically determined metabolic capacity. Our analysis crystallizes three pivotal lessons: the critical risk of SSRI and MAO-B inhibitor combinations in CYP2D6 intermediate metabolizers; the peril of “therapeutic inertia” caused by inadequate washout periods; and the imperative for pre-emptive pharmacogenetic screening and careful consideration of all potential drug-drug interactions in complex therapeutic regimens.

The pathogenesis of SS in this case is a quintessential example of a pharmacodynamic synergy unleashed by a multifactorial pharmacokinetic perfect storm. While the combination of SSRIs and MAO-B inhibitors is recognized as risky, its lethality in this patient was likely driven by a combination of factors, with the CYP2D6 *10/*10 intermediate metabolizer phenotype playing a significant role. However, it is crucial to avoid overstating its role. Sertraline is not primarily metabolized by CYP2D6; its major metabolic pathways involve CYP2B6, CYP2C19, and CYP3A4 (6). The patient’s *CYP2C19* intermediate metabolizer status may have further contributed to reduced sertraline clearance. Escitalopram is a substrate for CYP2C19, CYP2D6, and CYP3A4 (7). A critical and previously overlooked aspect is the role of CYP3A4. The patient was receiving multiple drugs that are substrates and/or inhibitors of CYP3A4, including quetiapine (major substrate), rivaroxaban (substrate), nifedipine (substrate). This polypharmacy likely created a significant competitive inhibition at the CYP3A4 enzyme, further impairing the metabolism of both sertraline and escitalopram and contributing to their toxic accumulation. This complex interplay of multiple inhibited metabolic pathways, rather than a single gene defect, more plausibly explains the extreme and rapid drug accumulation. While therapeutic drug monitoring was not performed to confirm this hypothesis, the pharmacological plausibility is strong. The resultant dramatic accumulation of both SSRIs created a massive serotonergic burden. Furthermore, rasagiline, while a selective MAO-B inhibitor, is not pharmacologically inert in this context. It and its metabolite are known to exhibit weak inhibition of CYP2D6, creating a vicious cycle of metabolic competition that further impeded the clearance of the offending SSRIs. Although the inhibitory effect of rasagiline on CYP2D6 is generally considered weak and clinically insignificant in most patients, in the context of a genetically compromised metabolic pathway (CYP2D6 *10/*10) and multiple competing substrates, even a weak inhibitor may contribute to a functionally relevant cumulative effect. This iatrogenic cascade transformed a standard-dose antidepressant trial into a relentless serotonergic storm. The subsequent transition from sertraline to escitalopram without a sufficient washout period represented a “second-hit” insult. The last dose of sertraline was administered on the evening of October 17, and the first dose of escitalopram was given on the morning of October 19, resulting in a washout period of approximately 36 hours. Given sertraline’s long elimination half-life (~26 hours)—which can extend significantly in intermediate or poor metabolizers—and the even longer half-life of its active metabolite, N-desmethylsertraline (~62–104 hours), a washout period of several weeks was warranted. The immediate introduction of escitalopram, itself susceptible to impaired clearance via the patient’s intermediate CYP2C19*1/*2 phenotype, led to a rapid, additive serotonergic surge, overwhelming neurological homeostasis.

The decision to initiate a second SSRI so rapidly after a probable episode of SS requires careful examination. While the patient’s HAMD-17 score of 19 indicated moderate depression and there may have been clinical urgency, the rationale for choosing another SSRI over a non-serotonergic alternative (e.g., mirtazapine) or optimizing the existing rasagiline therapy is not documented and represents a significant deviation from safe prescribing practices. This decision was made despite the known interaction risk and the recent adverse event, highlighting a critical gap in the clinical decision-making process. The absence of a washout period and the use of a standard 10 mg starting dose of escitalopram in an elderly, sensitive patient further compounded the risk. Notably, the medical records do not indicate whether a lower geriatric dose (e.g., 2.5 mg or 5 mg) was considered. Several factors may explain this omission. First, in busy hospital settings, consultation notes may not always explicitly document every alternative considered, particularly when the chosen dose falls within standard prescribing ranges. Second, while geriatric dose adjustment is recommended for many psychotropic medications, awareness of this principle may vary across providers and clinical contexts. Third, and most significantly, the prescribing team may not have fully appreciated the cumulative impact of this patient’s multiple risk factors: advanced age, CYP2D6 intermediate metabolizer status, concomitant use of a CYP2D6 inhibitor (rasagiline), and concurrent administration of multiple CYP3A4 substrates—all of which could substantially elevate escitalopram concentrations even at standard starting doses.

This oversight further reinforces the need for systematic approaches to geriatric prescribing that do not rely solely on individual prescriber vigilance. In retrospect, the assumption that a ‘standard’ dose would be appropriate for this patient—despite his age, genetic profile, and complex medication regimen—reflects a cognitive blind spot that is all too common in clinical practice. The prescribing team likely focused on the immediate therapeutic goal (treating severe depression) without fully integrating the accumulating risk factors that made this patient exceptionally vulnerable.

This case thus serves as a powerful reminder that standard adult dosing algorithms may not be appropriate for complex elderly patients with multiple interacting medications and genetic vulnerabilities. It illustrates a critical systems vulnerability: the absence of routine, automated alerts or decision support tools that integrate pharmacogenetic risk, drug-drug interactions, and age-related considerations at the point of prescribing. Such tools could prompt clinicians to consider dose reduction not as an exception, but as the default approach in complex, elderly patients. Until such systems are widely implemented, the burden falls on individual clinicians to maintain a high index of suspicion and to explicitly document their consideration of dose adjustment—a responsibility that this case demonstrates is easily overlooked in busy clinical environments.

Beyond the dose selection itself, another equally critical oversight occurred earlier in the clinical timeline. An additional critical oversight warrants careful examination: despite a documented adverse reaction to sertraline (tremor and agitation) just five months prior, this agent was re-initiated in October 2025. In retrospect, the May 2025 symptoms—mild tremor and agitation—were subtle and overlapped considerably with manifestations of Parkinson’s disease itself; at the time, they were attributed to motor fluctuations rather than pharmacologically induced serotonergic hyperactivity, particularly given the patient’s long history of uneventful sertraline use years earlier. This ambiguity is precisely what makes this case so instructive: in complex patients with underlying neurological disease, prodromal drug toxicity signals can be easily masked by the clinical features of the condition being treated. The medical records do not clearly document whether the May event was reviewed during the psychiatric consultation; we believe the most likely explanation is that this information was not effectively communicated between care settings—an increasingly common challenge when patients receive care from multiple providers across different healthcare systems. However, even if the event had been reviewed, its nonspecific nature may have reasonably been judged insufficient to preclude re-challenge, especially in the context of severe, debilitating depression and the patient’s prior long-term tolerability of sertraline. This case thus highlights not a simple error, but a fundamental diagnostic challenge at the intersection of neurology and psychiatry: distinguishing disease progression from drug toxicity when both can present with overlapping symptomatology.

Collectively, these observations underscore several critical lessons for safe prescribing. First, a patient’s drug history must be understood not merely as a list of prior exposures but as a record of prior biological responses that may predict future vulnerability—but this requires recognizing that such responses may be obscured by the patient’s underlying disease. Second, systematic review of prior adverse drug reactions should be a mandatory component of any medication reconciliation process, yet clinicians must remain aware that adverse reactions in neurologically complex patients may present atypically and require a high index of suspicion. Third, in complex polypharmacy scenarios, consultation notes should explicitly document review of prior tolerability data and the rationale for therapeutic decisions, including consideration of diagnostic uncertainty. Finally, this case highlights that communication failures between care providers represent a fundamental patient safety risk—but so does the inherent difficulty of interpreting ambiguous symptoms in patients with chronic neurological conditions, and both warrant systematic attention at the institutional and educational levels.

This case report is distinguished from the majority of published SS cases by its unique triad of features. First, it illustrates a sequential, dual-SSRI challenge superimposed on a background of MAO-B inhibition and extensive polypharmacy affecting multiple CYP enzymes, a scenario far more complex than the single-drug interactions or overdoses commonly reported (8). Second, the resultant multi-organ failure was of exceptional severity, progressing to rigid-coma, hyperpyrexia (>42 °C), and a disseminated intravascular coagulation (DIC)-like syndrome. This extreme presentation is directly attributable to the sustained toxic exposure enabled by the delayed drug clearance caused by the convergence of genetic polymorphisms and multiple drug-drug interactions. Third, this case critically challenges the perceived safety profile of selective MAO-B inhibitors. While fatalities are well-documented with irreversible, non-selective MAOIs (4), rasagiline is often considered a lower-risk agent. Our report provides compelling evidence that even selective MAO-B inhibitors can act as fulminant agents of SS when partnered with SSRIs in a poly-medicated, genetically susceptible host, fundamentally altering their risk-benefit calculus in such populations.

The diagnostic odyssey herein offers critical insights. The prodromal symptoms of tremor and labile hypertension were nonspecific and could easily be misattributed to Parkinson’s disease motor fluctuations or akathisia from antipsychotic medication (9). This underscores the imperative for neurologists and psychiatrists to maintain a high index of suspicion for incipient SS when introducing serotonergic agents in complex patients. The normal serum sodium levels helped exclude another common SSRI-related adverse effect. The role of quetiapine presents a clinical paradox. Although its 5-HT2A antagonist properties are theorized to be protective in SS, quetiapine is itself a minor substrate for CYP2D6 and a major substrate for CYP3A4 (10). In this patient, its accumulation—potentially exacerbated by the same CYP3A4 competition—likely contributed to overall sedation and toxicity, potentially negating any modest receptor-level benefits and illustrating the perils of relying on a drug’s theoretical pharmacodynamics without considering its real-world pharmacokinetics in a specific patient. The administration of cyproheptadine, a potent 5-HT2A antagonist, aligns with established treatment guidelines for severe SS and its use was a rational intervention in this critical setting.

This tragic outcome serves as a powerful mandate for the integration of pharmacogenomics into standard clinical practice, but also for a more holistic approach to assessing drug-drug interactions. We strongly advocate for pre-emptive *CYP2D6*, *CYP2C19*, and, when indicated, CYP3A4 phenotype assessment (through genotyping or phenotyping) in patients with Parkinson’s disease and other complex neurological conditions where polypharmacy is the norm and the use of psychotropic agents is likely. For identified intermediate or poor metabolizers, SSRIs should be either avoided in favor of non-serotonergic alternatives (e.g., mirtazapine) or initiated with extreme caution, conservative dosing, and enforced prolonged washout periods during any transitions. Moreover, a systematic review of a patient’s entire medication profile for potential metabolic competitions (e.g., at CYP3A4) is essential before initiating any new therapy with a narrow therapeutic index.

Our study is not without limitations. The lack of serial drug concentration monitoring precludes a definitive pharmacokinetic model of the accumulation. The net clinical effect of quetiapine remains ambiguous. Lastly, the specific contribution of the identified genetic and drug interaction factors, while mechanistically plausible, requires validation in larger pharmacoepidemiological studies.

In conclusion, this case is a sobering reminder that a patient’s genetic makeup and overall medication burden can irrevocably override conventional drug safety profiles. It argues compellingly for a paradigm shift towards comprehensively genotype-guided personalized medicine, where pre-emptive genetic data and a thorough analysis of all potential drug-drug interactions inform safer therapeutic choices and prevent such devastating therapeutic misadventures.

## Data Availability

The raw data supporting the conclusions of this article will be made available by the authors, without undue reservation.
