# Supplementary material for: Case Report: Pharmacogenetically-triggered fatal serotonin syndrome following sequential serotonergic therapy in Parkinson’s disease: a case highlighting the role of CYP2D6*10/*10 genotype and multifactorial drug interactions
Source: Front Psychiatry. 2026 Mar 31;17:1750596. doi: 10.3389/fpsyt.2026.1750596 (PMC13076281; doi:10.3389/fpsyt.2026.1750596)
Supplement: Supplementary file 1 [file Table1.docx]

### ****Supplementary Table S1. Application of the Hunter Serotonin Toxicity Criteria to the Presented Case****

| ****Criterion**** | ****Presence in Patient (Yes/No)**** | ****Clinical Findings in This Case Supporting the Criterion**** | Notes/Limitations |
| --- | --- | --- | --- |
| **Exposure to a serotonergic agent** | Yes | Patient received sertraline (50 mg × 2 doses) followed by escitalopram (10 mg × 3 doses, as the morning dose on Oct 19 was administered) while on stable rasagiline (MAO-B inhibitor) therapy. | Clear temporal relationship established. The last dose of sertraline was on Oct 17 evening; the first dose of escitalopram was on Oct 19 morning. |
| **Spontaneous clonus** | Unable to assess | The patient exhibited myoclonus, which is a recognized sign of neuromuscular hyperexcitability in serotonin syndrome. However, spontaneous clonus was not documented. | While myoclonus supports the diagnosis of serotonin toxicity, it does not fulfill the specific "spontaneous clonus" criterion of the Hunter Criteria. The diagnosis is definitively established through the Tremor + Hyperreflexia criterion. |
| **Inducible clonus** + agitation/diaphoresis | **Unable to assess** | Patient's critical condition (rigid-coma) precluded formal testing for inducible clonus. Agitation was present prior to coma. | Clinical status limited full neurological examination during fulminant phase. |
| **Ocular clonus** + agitation/diaphoresis | **Unable to assess** | Ocular clonus was not specifically documented. Anisocoria was present, indicating brainstem involvement. Agitation was present prior to coma. | Ocular clonus requires specific examination; not documented in available records. |
| **Tremor** + **hyperreflexia** | Yes | Coarse tremor was documented. Patellar and Achilles hyperreflexia (3+) was noted on neurological examination. | This combination alone is sufficient to meet Hunter Criteria for the diagnosis of serotonin toxicity. |
| **Hypertonia** + temperature >38°C + **ocular clonus**/inducible clonus | No | The patient exhibited increased muscle tone (hypertonia) and core temperature >38°C (peak 42°C). However, as noted above, neither ocular nor inducible clonus could be assessed. | This specific triad could not be fulfilled due to the inability to assess for clonus, but the presence of hypertonia and hyperthermia in this context is highly suggestive of severe serotonin toxicity. |
| **Overall Diagnosis** | **Yes** | The patient definitively meets the Hunter Serotonin Toxicity Criteria based on the combination of Tremor + Hyperreflexia. | The patient definitively meets the Hunter Serotonin Toxicity Criteria through the Tremor + Hyperreflexia pathway, as documented on neurological examination. The overall clinical context—including myoclonus, hyperthermia (peak 42°C), autonomic instability, and altered mental status—further supports the diagnosis of severe, life-threatening serotonin syndrome. |
